# Efficacy of Cannabidiol for Δ-9-Tetrahydrocannabinol-Induced Psychotic Symptoms, Schizophrenia, and Cannabis Use Disorders: A Narrative Review

**DOI:** 10.3390/jcm10061303

**Published:** 2021-03-22

**Authors:** Francesco Bartoli, Ilaria Riboldi, Bianca Bachi, Angela Calabrese, Federico Moretti, Cristina Crocamo, Giuseppe Carrà

**Affiliations:** 1 Department of Medicine and Surgery, University of Milano Bicocca, via Cadore 48, 20900 Monza, Italy; i.riboldi1@campus.unimib.it (I.R.); b.bachi@campus.unimib.it (B.B.); a.calabrese16@campus.unimib.it (A.C.); f.moretti24@campus.unimib.it (F.M.); cristina.crocamo@unimib.it (C.C.); 2 Division of Psychiatry, University College London, Maple House 149, London W1T 7BN, UK

**Keywords:** Cannabidiol, Δ-9-tetrahydrocannabinol, schizophrenia, psychotic disorders, cannabis use disorder, dual diagnosis

## Abstract

Although cannabis’ major psychoactive component, Δ-9-tetrahydrocannabinol (THC), has been linked to both earlier onset and poorer outcomes of psychotic disorders, Cannabidiol (CBD) seems to have different pharmacological mechanisms and potential therapeutic properties. However, no clinical study has investigated CBD for the treatment of co-occurring psychotic and cannabis use disorders so far, even though its utility seems grounded in a plausible biological basis. The aim of this work is thus to provide an overview of available clinical studies evaluating the efficacy of CBD for psychotic symptoms induced by THC, schizophrenia, and cannabis use disorders. After searching for relevant studies in PubMed, Cochrane Library, and ClinicalTrials.gov, we included 10 clinical studies. Available evidence suggests that CBD may attenuate both psychotic-like symptoms induced by THC in healthy volunteers and positive symptoms in individuals with schizophrenia. In addition, preliminary data on the efficacy of CBD for cannabis use disorders show mixed findings. Evidence from ongoing clinical studies will provide insight into the possible role of CBD for treating psychotic and cannabis use disorders.

## 1. Introduction

Over 192 million individuals aged 15–64 years used cannabis during 2018, corresponding to 3.9% of the global population [1]. In Europe, this was true for 7.6% of the general population, who had used cannabis at some time in their life, whilst 15.0% of individuals aged under 24 used cannabis in the last year [2]. Data from the U.S. National Surveys on Drug Use and Health (NSDUH), based on 749,500 adults, showed that the prevalence of cannabis use disorder in the past year is around 1–2% [3]. In addition, substance use disorders are frequently comorbid conditions in severe mental illnesses [4,5], with cannabis being the most used illicit substance [6,7]. Nevertheless, interest in the therapeutic role of cannabis has grown in the last few years, based also on the recent changes regarding legal access to cannabis for therapeutic purposes [8] and on the potential use of cannabis for mental disorders [9,10,11]. The biochemical complexity of cannabis, with a combination of hundreds of different chemovars, including cannabinoids, flavonoids, and terpenoids, rather than a single-agent compound [12], may partly explain the relevant treatment rationale. Among cannabinoids isolated in cannabis, two are of greater interest in mental healthcare, i.e., Δ-9-tetrahydrocannabinol (THC) and Cannabidiol (CBD) [11]. Previous studies pointed out that the relationship between cannabis and psychotic symptoms might be explained by its potency, namely the relative content of THC as compared with CBD [13,14,15]. Whereas THC may induce psychotic and other psychiatric symptoms with large effect sizes [16], it seems that CBD may alleviate the effects of THC on the human brain. An interaction between CBD and THC has in fact been highlighted since the 1970s, when the first experimental studies on healthy volunteers showed that CBD might attenuate neuropsychological and behavioral effects induced by THC [17,18,19]. Early case series [20,21] highlighted the potential antipsychotic effects of CBD. More recently, preliminary data provided initial evidence that CBD may improve psychotic symptoms, while also possibly being better tolerated than standard antipsychotic medications [22,23]. It seems that, as compared with THC, CBD may have antipsychotic and anxiolytic properties, without detrimental effects on cognition [24]. A previous systematic review [25] tried to summarize findings from clinical studies and case reports on CBD as a treatment for psychosis and addictive behaviors. The authors emphasized the promising role of CBD, considering that a dysregulation of the endocannabinoid system might be involved in both disorders. However, among different addictive behaviors, specific effects of CBD for comorbid cannabis use disorders remain to be clarified. This issue is crucial considering the high rates of cannabis use disorders among individuals with schizophrenia [26], especially those with first-episode psychosis [27]. Even though there is a lack of evidence on standard antipsychotic medications for subjects with both psychotic and cannabis use disorders [28,29,30], no clinical study has investigated CBD for the treatment of these co-occurring disorders. Nonetheless, it is important to analyze potential alternative pharmacological approaches with a strong biological rationale such as CBD. In this narrative review, we summarized the available findings on CBD for THC-induced psychotic symptoms, schizophrenia, and cannabis use disorders. 

## 2. Materials and Methods

This review was conducted following the Scale for the Assessment of Narrative Review Articles (SANRA) items [31]. We included clinical studies testing the efficacy of CBD in (i) healthy volunteers after the administration of THC; (ii) subjects suffering from schizophrenia; (iii) individuals with cannabis use disorders. We searched PubMed and the Cochrane Library for trials published up to January 2021. ClinicalTrials.gov was explored for clinical trials that were completed yet unpublished, with reported findings. Our search strategy combined the following terms: “cannabidiol” AND (“cannabis” OR “schizophrenia”). An additional screening of the studies included in two recent, relevant reviews was made [24,25]. 

## 3. Results

### 3.1. Study Selection 

Our searches generated 108, 328, and 120 trials from PubMed, the Cochrane Library, and ClinicalTrials.gov, respectively. In addition, 16 and 10 studies were retrieved through screening the reference lists of two recent reviews [24,25]. We identified 10 eligible studies, i.e., four studies [32,33,34,35] exploring the effects of CBD in healthy volunteers taking THC; four studies [36,37,38,39] testing CBD in people with schizophrenia; and two studies [40,41] investigating CBD for cannabis use disorders. No clinical trial testing CBD in subjects with both psychotic and cannabis use disorders was identified.

### 3.2. Effects of Cannabidiol on Δ-9-tetrahydrocannabinol-Induced Psychotic Symptoms in Healthy Volunteers

Data from four clinical studies testing the effects of CBD on THC-induced psychotic symptoms were available (Table 1). The first study was published in 2010 [32]. The authors evaluated whether a pre-treatment with CBD could prevent the occurrence of acute psychotic symptoms induced by THC in six healthy volunteers reporting lifetime cannabis use. Using a double-blind design, CBD (5 mg) or placebo was administered intravenously immediately before THC (1.25 mg). Positive psychotic symptoms were assessed according to the Positive and Negative Syndrome Scale (PANSS) at baseline, as well as 30 and 90 min after THC intravenous administration. Despite the limited sample size, the authors found that pre-treatment with CBD decreased the occurrence of psychotic symptoms assessed 30 min after the administration of THC, lowering the PANSS’ positive score from 13 (±5.8) to 9 (±2.2).

In a subsequent study, Englund et al. [33] tested the hypothesis that pre-treatment with CBD could inhibit THC-induced psychosis. Healthy participants reporting lifetime cannabis use were randomized to receive oral CBD (600 mg; *n* = 22) or placebo (*n* = 26) 210 min before intravenous THC (1.5 mg over 10 min). The CBD group showed lower PANSS positive scores after THC administration, although this difference was not statistically significant. In addition, the occurrence of clinically meaningful, positive symptoms (defined as increases ≥ 3 points) was less likely in the CBD group as compared with the placebo group (OR: 0.22; *p* < 0.05). Consistently, individuals from the CBD group, compared with those treated with the placebo, were less likely to have post-THC paranoia.

However, contradictory findings were provided by the study of Mueller et al. [35], even though, to our knowledge, data were shown only in a conference abstract. In this randomized, double-blind, placebo-controlled clinical trial, the healthy volunteers (*n* = 60) were randomly allocated to four different treatments, i.e., THC (20 mg orally), CBD (800 mg orally), CBD + THC, and placebo. While CBD alone did not induce any psychotic features, it also did not provide any protective effects against THC-induced psychotic symptoms. Indeed, higher PANSS total and positive scores were estimated among individuals treated with both THC/placebo and THC/CBD, as compared with those treated with CBD/placebo or placebo/placebo. 

Finally, there were mixed results shown in a subsequent randomized, double-blind study, based on a crossover design [34], that compared the effects of inhaled THC (8 mg), CBD (16 mg), THC + CBD (8 + 16 mg), and placebo, in 48 volunteers reporting cannabis use. Findings showed that THC increased both psychotomimetic symptoms, as measured by the Psychotomimetic States Inventory, and negative symptoms, assessed according to the Brief Psychiatric Rating Scale. Nonetheless, the co-administration of CBD did not alleviate these effects, whereas CBD alone reduced the Psychotomimetic States Inventory scores in light users, but not in heavy ones.

### 3.3. Efficacy of Cannabidiol for Schizophrenia Spectrum Disorders

Four clinical trials tested CBD in people with schizophrenia. Relevant findings are summarized in Table 2.

The first clinical trial, published in 2010 [37], tested the cognitive performance of 28 individuals suffering from schizophrenia, receiving either a single dose of CBD (300 mg, *n* = 9, or 600 mg, *n* = 9) or placebo (*n* = 10). The authors found that a single administration of CBD had no beneficial acute effects on selective attention during the performance of the Stroop Color Word Test, although the hypothesis that chronic treatment might lead to an improvement could not be excluded.

The lack of the effects of CBD on cognition was confirmed by a subsequent study published in 2018 by Boggs and colleagues [36]. In this 6-week, randomized, placebo-controlled, parallel-group, fixed-dose trial, oral CBD (600 mg, *n* = 18) and placebo (*n* = 18) augmentation were compared in 36 clinically stable outpatients with chronic schizophrenia who were routinely treated with antipsychotics. The authors estimated that CBD augmentation, despite being well tolerated, was not associated with an improvement in the MATRICS Consensus Cognitive Battery.

The first and only head-to-head clinical trial, comparing CBD with amisulpride monotherapy, was published in 2012 [38]. In this 4-week double-blinded, randomized, parallel-group, controlled trial, 42 individuals with schizophrenia were randomized (1:1) to receive either CBD or amisulpride up to a daily dose of 800 mg within the first week. Subjects treated with CBD and those treated with amisulpride showed a comparable clinical improvement, as assessed by the decrease in the PANSS’ total scores and subdomains, i.e., positive, negative, and general psychopathology scores. Moreover, in terms of the proportion of responders (≥20% improvement in the PANSS’ total score), no differences between treatments were estimated (CBD 15/20, amisulpride 14/19). Thus, these results suggest the possible non-inferiority of CBD as compared with amisulpride. Moreover, CBD was well tolerated and associated with fewer extrapyramidal symptoms, lower weight gain, and prolactin increase, without affecting hepatic or cardiac functions.

Finally, in a phase 2, 8-week, multicenter, double-blind, randomized, placebo-controlled, parallel-group trial [39], individuals with schizophrenia were randomized (1:1) to receive CBD (1000 mg; *n* = 43) or placebo (*n* = 45) as an adjunctive treatment to antipsychotics. After 6 weeks, the individuals treated with CBD showed a significant decrease in PANSS positive symptoms (–3.2 ± 2.6) as compared with placebo (–1.7 ± 2.8), with a treatment difference of −1.4 (95% CI= −2.5 to −0.2), corresponding to an estimated moderate effect size of 0.56. No differences in the PANSS’ total and negative symptoms were found. In addition, subjects taking CBD were more likely to have been rated as “improved” and “not severely unwell”, according to the Clinical Global Impressions scale. Finally, data confirmed that CBD was well tolerated, showing rates of adverse events comparable to those of placebo treatment.

### 3.4. Efficacy of Cannabidiol for Cannabis Use Disorders

Two clinical trials—one published [40] and another unpublished, with results reported on ClinicalTrials.gov (accessed on 20 January 2021) [41]—tested CBD for cannabis use disorders. Relevant findings are summarized in Table 3.

Freeman et al. [40] conducted a phase 2a, double-blind, placebo-controlled, randomized, adaptive Bayesian trial. In this trial, forty-eight individuals with DSM-5 cannabis use disorder, after a brief psychological motivational intervention, were randomly assigned to a 4-week treatment with placebo (*n* = 12), CBD 200 mg (*n* = 12), CBD 400 mg (*n* = 12), and CBD 800 mg (*n* = 12). The group receiving CBD 200 mg was dropped from the trial due to lack of response, and 34 additional participants were allocated to CBD 400 mg (*n* = 12), CBD 800 mg (*n* = 11), and placebo (*n* = 11). 

The urinary 11-nor-9-carboxy-δ-9-tetrahydrocannabinol (THC-COOH)/creatinine ratio, as a measure of recent cannabis use, and the number of days abstinent from cannabis, were the primary outcomes. Individuals treated with CBD 400 mg had a decreased urinary THC-COOH/creatinine ratio by −94.2 ng/mL (95% CI: −161.8 to −35.6) and increased abstinence from cannabis by 0.48 days per week (95% CI: 0.15 to 0.82), as compared with those treated with the placebo. In addition, CBD 800 mg decreased the THC-COOH/creatinine ratio by −72.0 ng/mL (95% CI: −135.5 to −19.5) and increased abstinence from cannabis by 0.27 days per week (95% CI: −0.09 to 0.64). The treatment completion rate was 94% (77/82 participants) and no severe adverse events were recorded.

Another clinical trial [41], based on 10 individuals (five receiving CBD up to 800 mg and five receiving placebo over a 6-week treatment period), was registered on ClinicalTrials.gov (accessed on 20 January 2021). Available data showed negative results for the primary outcome, i.e., self-reported cannabis inhalations per day during week 6, as reported by the Timeline Follow-Back, an instrument to measure daily cannabis use. Indeed, subjects treated with CBD had a higher mean number of inhalations of cannabis per day as compared with patients receiving a placebo (26.7 ± 27.0 vs. 5.3 ± 4.3).

## 4. Discussion

This comprehensive review synthesized major findings on CBD in human studies for psychotic and cannabis use disorders. We reported evidence from clinical studies analyzing the potential effects of CBD on psychotic symptoms induced by THC, from trials testing the efficacy of CBD for psychotic disorders, and from others investigating the effects of CBD for cannabis use disorders. 

Several findings can be derived from this narrative review. First, available data seem to indicate that CBD might attenuate the acute psychotic effects induced by THC in non-clinical samples. However, additional research is needed, considering the limited sample size of studies, the methodological variability, and the inconsistency of results, which do not allow one to draw firm conclusions. The beneficial acute effect appears to have a greater magnitude when CBD is administered immediately before THC, without a latency period, and with intravenous, rather than oral, administration. Second, current evidence does not seem to support any effects of CBD on cognition in people with schizophrenia [36,37]. Nonetheless, in trials testing 800 mg [38] and 1000 mg [39], CBD seemed to be potentially effective in reducing the positive symptoms of schizophrenia, as both standalone and add-on treatment to antipsychotics. However, no efficacy on positive symptoms at a lower dosage (600 mg) [36] and no effects on negative symptoms have been observed. As regards the use of CBD for improving abstinence from cannabis use, evidence, though promising, is mixed. A recent trial [40], based on a substantial pool of individuals, suggested the efficacy and safety of CBD for cannabis use disorders, counterbalancing the negative findings from a previous unpublished trial [41] based on a smaller sample. Nonetheless, this was a phase 2a trial [40], not designed to estimate the magnitude of efficacy. Any inferences about CBD clinical relevance and related recommendations would thus be premature [42]. Moreover, additional data on CBD optimal dosing and timing of treatment are needed, also considering specific individual factors that may influence its efficacy, such as the individual degree of motivation to change and the duration of cannabis use disorder [43].

Taken together, this preliminary, circumstantial evidence provides additional insight into CBD as a promising treatment for THC-induced psychotic symptoms and schizophrenia.

Established neurobiological mechanisms add plausibility to the likely efficacy of CBD. First, it should be noted that THC and CBD have different, and somehow opposite, actions on receptors of the endocannabinoid system, namely CB1 and CB2. CB1 receptors are highly expressed in the basal ganglia nuclei, hippocampus, cortex, and cerebellum, being primarily located on the terminals of central and peripheral neurons, as well as in astrocytes, though at much lower levels than in neurons. Conversely, CB2 receptors are mainly expressed in peripheral organs with immune function [44]. While THC is a partial agonist of the cannabinoid CB1 receptors, CBD is a negative allosteric modulator and could inhibit the cannabinoid agonist activity [45]. The evidence that CBD does not activate the receptor directly, but actually alters the potency and efficacy of CB1 ligands, supports the hypothesis that CBD might antagonize some of the effects of THC, as reported in clinical studies [46]. Second, the possible involvement of the endocannabinoid system in schizophrenia spectrum disorders has been highlighted in a recent systematic review and meta-analysis [47], reporting higher central and peripheral levels of anandamide, an endogenous cannabinoid, among individuals with schizophrenia. It has been hypothesized that cannabis use might have an impact on mental health, altering endocannabinoid signaling in the central nervous systems of individuals suffering from schizophrenia [48]. On the other hand, CBD, inhibiting the enzyme fatty acid amide hydrolase, may in turn enhance anandamide signaling [22]. Consistently, an association between the increase in anandamide blood levels and the decrease in psychotic symptoms in subjects treated with CBD has been reported [38]. Finally, it has been hypothesized that the contrasting effects induced by THC and CBD on regional brain function may underlie their different behavioral effects [49]. Bhattacharyya et al. [50] showed that THC and CBD have opposite effects in the striatum during verbal recall, in the hippocampus during the response inhibition task, in the amygdala when subjects view fearful faces, and in the superior temporal cortex when subjects listen to speech, as well as in the occipital cortex during visual processing. A systematic review and meta-analysis of neuroimaging studies [51], based on 24 human and 21 animal studies, tested the acute effects of cannabinoids on brain functioning. Relevant findings, though based on a high degree of methodological variability, highlighted that THC and CBD have opposite neurophysiological effects. The complex relationship between the neurobiological underpinnings of the endocannabinoid system and psychosis remains to be elucidated. For example, abnormalities in immune response have been found among individuals with first-episode psychosis [52], and it has been consistently hypothesized that exposure to cannabis, especially during adolescence, might cause immunological dysfunctions that may in turn induce a latent vulnerability to psychosis [53]. Interestingly, it has been hypothesized that the potential neuroprotective and anti-inflammatory properties of CBD, mediated by the inhibition of microglial activation, might explain at least a portion of its antipsychotic effects [24,54]. 

Although clinical trials seem to highlight CBD as a promising treatment for psychosis, additional studies are needed before claiming its efficacy and acceptability. There are several ongoing trials, with protocols registered in ClinicalTrials.gov, whose results are still not available, namely (i) a 4-week trial investigating the efficacy and safety of CBD (400 mg), as compared with both olanzapine (15mg) and placebo, for acutely ill individuals with schizophrenia [55]; (ii) an 8-week trial testing CBD (500 mg for 1 week, then 1000 mg) in early psychosis [56]; (iii) a crossover trial testing CBD (800 mg) for schizophrenia, investigating the effects of CBD followed by placebo and placebo followed by CBD [57]; (iv) a 26-week trial evaluating CBD (400 mg) as an adjunctive drug for maintenance treatment of schizophrenia [58]. More interestingly, two ongoing trials purposively aim to test the efficacy and the tolerability of CBD for comorbid psychotic and cannabis use disorders: (i) a 6-week trial comparing the effect of a monotherapy with CBD (600 mg) and with risperidone (4 mg) for treating individuals with both psychosis and cannabis use disorder [59]; (ii) a 12-week trial evaluating CBD (600 mg) on psychiatric symptoms, cognition, and cannabis consumption in regular cannabis users with recent-onset psychosis [60]. Certainly, in the next few years, findings from these clinical studies will provide additional insight into the potential role of CBD for schizophrenia and cannabis use disorders. These results could also be useful in defining the target dose and ideal length of CBD treatment for these conditions, given the heterogeneous dosages ranging between 200 and 1000 mg and the different treatment durations tested so far. In addition, considering the high variability in the bioavailability of CBD, depending on the route of administration, future research should clarify which are the best treatment approaches for CBD [61].

## 5. Conclusions

According to preliminary evidence showing its impact on alleviating THC-induced psychotic symptoms and its possible efficacy for schizophrenia, CBD might be considered a promising therapeutic option for psychotic disorders. Nonetheless, the relationship between the endocannabinoid system and neurobiological abnormalities, possibly associated with psychosis, needs to be better explored. On the other hand, mixed results can be derived from available trials investigating CBD for cannabis use disorders. Although the utility of CBD seems grounded in a plausible biological foundation, additional evidence is needed, possibly overcoming the heterogeneity of previous clinical studies in terms of assessment measures, concomitant treatments, and target doses. Several clinical trials are ongoing and will also provide insight into the possible role of CBD for treating co-occurring psychotic and cannabis use disorders.

## Figures and Tables

**Table 1:** Clinical studies testing the acute effects of Cannabidiol on Δ-9-tetrahydrocannabinol-induced psychotic symptoms in healthy volunteers.

Study	Study Design	Participants	Intervention	CBD Administration	Results
Bhattacharyya et al., 2010 [32]	Double-blind, within-subject, pseudorandomized, placebo-controlled trial	6 healthy volunteers with lifetime cannabis use	CBD ^1^ 5 min before THC ^2^	Intravenous; 5 mg	PANSS ^3^ positive scores lower in the CBD pre-treatment group.
Englund et al., 2013 [33]	Double-blind, randomized, placebo-controlled trial	48 healthy volunteers with lifetime cannabis use	CBD 210 min before THC	Oral; 600 mg	No differences in PANSS positive scores; lower frequency of clinically significant positive symptoms as measured by PANSS in the CBD group; lower SSPS ^4^ scores in the CBD group.
Morgan et al., 2018 [34]	Double-blind, crossover, randomized, controlled trial	48 volunteers with cannabis use (24 light and 24 heavy users)	4 sessions in the same group: (i) THC; (ii) CBD; (iii) CBD+THC; (iv) placebo	Inhaled; 16 mg	No effects of CBD on THC-induced psychotomimetic symptoms as measured by the PSI ^5^; improvement in psychotomimetic symptoms in light users.
Mueller et al., 2016 [35]	Double-blind, placebo-controlled trial	60 healthy volunteers	4 groups: (i) THC/placebo; (ii) CBD/placebo; (iii) THC/CBD; (iv) placebo/placebo	Oral; 800 mg	Higher PANSS and APZ ^6^ scores in THC/placebo and THC/CBD groups as compared with those in CBD/placebo and placebo/placebo groups.

^1^ CBD: Cannabidiol; ^2^ THC: Δ-9-Tetrahydrocannabinol; ^3^ PANSS: Positive and Negative Syndrome Scale; ^4^ SSPS: State Social Paranoia Scale; ^5^ PSI: Psychotomimetic States Inventory; ^6^ APZ: Abnormer Psychischer Zustand.

**Table 2:** Clinical trials testing Cannabidiol as a treatment for schizophrenia spectrum disorders.

Study	Study Design	Participants	Intervention	CBD Administration	Follow-Up Time	Results
Boggs et al., 2018 [36]	Double-blind, placebo-controlled trial	36 outpatients with chronic schizophrenia	CBD ^1^ in adjunction to basic antipsychotic treatment	Oral; 600 mg/a day	6 weeks	CBD not associated with an improvement in PANSS ^2^ or MCCB ^3^ scores.
Hallak et al., 2010 [37]	Double-blind, placebo-controlled trial	28 outpatients with schizophrenia	Single dose of CBD	Oral; 300 mg or 600 mg		Better SCWT ^4^ performance in CBD 300-mg and placebo groups than in CBD 600-mg group.
Leweke et al., 2012 [38]	Double-blind, head-to-head, randomized trial (CBD vs. amisulpride)	39 inpatients with acute schizophrenia	CBD monotherapy	Oral; up to 800 mg/a day within the 1st week	4 weeks	Comparable clinical improvement in CBD and amisulpride for PANSS scores.
McGuire et al., 2018 [39]	Double-blind, placebo-controlled trial	88 outpatients with schizophrenia	CBD in adjunction to basic antipsychotic treatment	Oral; 1000 mg/a day	6 weeks	Reduction in PANSS positive scores in CBD group; no effects on cognition performance as measured by the BACS ^5^.

^1^ CBD: Cannabidiol; ^2^ PANSS: Positive and Negative Syndrome Scale; ^3^ MCCB: MATRICS Consensus Cognitive Battery; ^4^ SCWT: Stroop Color Word Test; ^5^ BACS: Brief Assessment of Cognition in Schizophrenia.

**Table 3:** Clinical trials testing Cannabidiol as a treatment for cannabis use disorder.

Study	Study Design	Participants	Intervention	CBD Administration	Follow-Up Time	Results
Freeman et al., 2020 [40]	Double-blind, randomized, placebo-controlled trial	82 outpatients with cannabis use disorder	CBD ^1^	Oral; 200 mg, 400 mg or 800 mg/a day	4 weeks	Reduction in 11-Nor-9-carboxy-δ-9-tetrahydrocannabinol/creatinine ratio and increased number of days of abstinence with CBD 400 mg and 800 mg.
NCT03102918 [41]	Double-blind, randomized, placebo-controlled trial	10 outpatients with cannabis use disorder	CBD	Oral; up to 800 mg/a day	6 weeks	Increased daily cannabis use in the CBD group.

^1^ CBD: Cannabidiol.
